# Mercury in traditionally foraged species of fungi (macromycetes) from the karst area across Yunnan province in China

**DOI:** 10.1007/s00253-020-10876-6

**Published:** 2020-09-21

**Authors:** Jerzy Falandysz, Małgorzata Mędyk, Martyna Saba, Ji Zhang, Yuanzhong Wang, Tao Li

**Affiliations:** 1 Environmental Chemistry and Ecotoxicology, University of Gdańsk, 80-308 Gdańsk, Poland; 2 Environmental and Computational Chemistry Group, School of Pharmaceutical Sciences, Zaragocilla Campus, University of Cartagena, Cartagena, 130015 Colombia; 3 Yunnan Academy of Agricultural Sciences, Medicinal Plants Research Institute, Kunming, 650200 Yunnan China; 4 Yuxi Normal University, School of Chemical Biology and Environment, Yuxi, 653100 Yunnan China

**Keywords:** Ethnobotany, Forest soil, Functional foods, *Ganoderma* spp., Traditional Asian medicines, Wild foods

## Abstract

**Abstract:**

The objective of this study is to better quantify the occurrence, intake, and potential risk from Hg in fungi traditionally foraged in SW China. The concentrations and intakes of Hg were measured from 42 species including a “hard” flesh type polypore fungi and a” soft” flesh type edible species that are used in traditional herbal medicine, collected during the period 2011–2017. Three profiles of forest topsoil from the Zhenyuan site in 2015 and Changning and Dulong sites in 2016 were also investigated. The concentrations of Hg in composite samples of polypore fungi were usually below 0.1 mg kg^−1^ dry weight (dw) but higher levels, 0.11 ± 0.01 and 0.24 ± 0.00 mg kg^−1^ dw, were noted in *Ganoderma applanatum* and *Amauroderma niger* respectively, both from the Nujiang site near the town of Lanping in NW Yunnan. Hg concentrations in *Boletaceae* species were usually well above 1.0 mg kg^−1^ dw and as high as 10 mg kg^−1^ dw. The quality of the mushrooms in this study in view of contamination with Hg showed a complex picture. The “worst case” estimations showed probable intake of Hg from 0.006 μg kg^−1^ body mass (bm) (“hard” type flesh) to 0.25 μg kg^−1^ bm (“soft” flesh) on a daily basis for capsulated products, from 17 to 83 μg kg^−1^ bm (“soft” flesh) in a meal (“hard” type flesh mushrooms are not cooked while used in traditional herbal medicine after processing), and from 0.042 to 1.7 and 120 to 580 μg kg^−1^ bm on a weekly basis, respectively.

**Graphical abstract:**

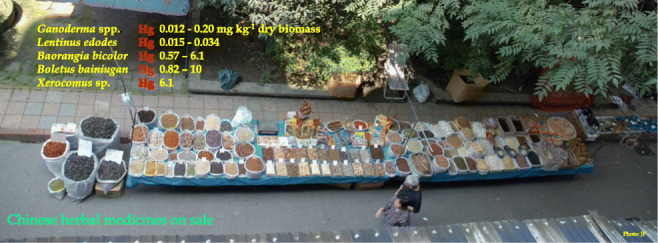

**Key points:**

• *Polypore species were slightly contaminated with Hg.*

• *Hg maximal content in the polypore was < 0.25 mg kg*^*−1*^ *dry weight.*

• *Many species from Boletaceae family in Yunnan showed elevated Hg.*

• *Locals who often eat Boletus may take Hg at a dose above the daily reference dose.*

## Introduction

Mushrooms (*Macromycetes*), dried and powdered or in the form of extracts, are traditionally popular in Chinese herbal medicine and elsewhere in Asia and are also considered functional foods (Bhatt et al. 2018; Wang et al. 2013; Wasser 2010). This traditional use of wild mushrooms as medicines has survived in other parts of the world, including Mexico (Nnorom et al. 2019; Santiago et al. 2016; Yongabi 2019). In the past, mushrooms were also used in folk medicine in Europe but this tradition has practically disappeared there (Gründemann et al. 2020; Grzywnowicz 2001, 2007). However, scientific evidence supporting the use of mushrooms in the treatment of disease is questioned or considered premature (Money 2016). Traditionally foraged edible species of fungi are valued in traditional herbal medicines largely due to active organic compounds and an example can be species from the genus *Fomitopsis* and *Ganoderma*, while much less is information on minerals and heavy metal contaminants (Bhatt et al. 2018; Nnorom et al. 2013; Zaidman et al. 2005).

Data shows that many mushrooms are vulnerable to contamination with mercury (Hg) compounds that are absorbed by the mycelia of the fungi and efficiently bioconcentrated in fruiting bodies both from the field and controlled experimental studies (Bressa et al. 1988; Crane et al. 2010; Falandysz et al. 2007). This characteristic has been associated to species-specific genetic features in connection to soil bedrock geochemical anomalies and anthropogenic pollution (Falandysz 2016; Melgar et al. 2009; Nasr and Arp 2011). The genetic, species-specific susceptibility of fungi to accumulation of Hg and their vulnerability to contamination with this element can be explained in part by the type and quantity of sulfur (S) and also selenium (Se)-containing ligands that they produce (Kavčič et al. 2018, 2019; Nasr et al. 2012). Mushrooms found in different regions of China can be substantially contaminated with geogenic Hg absorbed from background soil from the mineral belt (Falandysz et al. 2015b). Continental Asia, and also some other continents, have areas with bedrock and soils enriched in mercury that occurs largely in the mineral, cinnabar (HgS), from the vast region of the Circum-Pacific Mercuriferous Belt (Gustin et al. 1999). Cinnabar mining at the Lanmuchang site in Gùizhōu in southwestern China, and the use of HgS as red pigment vermilion and as a sedative in traditional herbal medicine, has an over 2000-year-old history in China (Wang 1944; Young et al. 2002).

Geogenic Hg is well dispersed on the land and oceans and due to degassing is subject to a specific global biogeochemical circulation (Ariya et al. 2015; Falandysz et al. 2020d). Regions with a mineral belt can be abundant not only in HgS, but also in ores bearing other elements such as As, F, Pb, Ag, Mo, Ni, Au, Re, Se, V, and Zn. The occurrence of Hg is also associated with anthropogenic activities such as copper-bearing shales (Kijewski 1989). Forest soils can act as a long-term sink of airborne Hg from anthropogenic emissions due to ongoing use of Hg and its compounds, legacy sources, gold mining, combustion of fossil fuels, and thermal processes and China is the major emitter of Hg from anthropogenic sources at both the regional and global scales (Chen et al. 2019; Suchara and Sucharova 2002).

Elevated and occasionally high concentrations of Hg in forest topsoils in several remote and rural areas in the Yunnan province in China strongly indicate the overriding role of geogenic sources of Hg in local mushrooms, but anthropogenic pollution in the forest topsoil is a source (in the 0-–10-cm layer of soil in Dayingjie in Yuxi up to 3.4 mg kg^−1^ dw) that cannot be neglected (Falandysz et al. 2015b). The organic layer of soil in forests efficiently retains airborne Hg from direct deposition, trough fall, and litter fall, and this makes a significant contribution to its absorption by mycelia and accumulation in the flesh of fruiting bodies (Demers et al. 2007; Falandysz et al. 2014). Mycelia are able to colonize and take up minerals from relatively large volumes of soil substrate including different layers of the soil horizon in which they grow. Some mushroom species also produce rhizomorphs and mycelial cords, which act as organs of absorption and translation of nutrients and water absorption and translocation, and colonization of substrates (Yafetto 2018). Therefore, the elemental contaminants contained in the mushroom’s fruiting bodies—regardless of the uptake, translocation, and accumulation mechanisms or competition between the elements—present an integral picture of the local environmental conditions in view of the physiology and adaptiveness of a particular species.

The Yunnan is home to a biological diversity of fungi species that grow within a vast mountainous terrain (Yunnan has area of 394,000 sq. km and the average elevation is 1980 m and a highest point has 6740 m a.s.l. at Kawagebo) that is characterized by canyons and large rivers (Nu, Yangzi, and Mekong rivers), as well as areas of polymetallic soils that are naturally enriched in As and Hg, and other hazardous elements to combined diversity in species and terrains present a challenge to the study of the local fungi (Li and Wang 2008; Wen and Chi 2007).

Additionally, knowledge and data on the occurrence of Hg in mushrooms that are popular in Chinese medicine are largely absent. Mercury and its compounds are highly toxic and especially relevant in foods including edible mushrooms is their contamination with neurotoxic mono-methylmercury (MeHg) (Fernandes et al. 2020; WHO 2011; Stijve and Roschnik 1974). It has to be emphasized that wild-growing mushrooms efficiently bioconcentrate Hg from soil currently contaminated with this element both due to geological processes and from anthropogenic depositions (Falandysz 2002, 2016; Kavčič et al. 2019; Nasr and Arp 2011; Saba et al. 2016a). In result, and due to high capacity of mushrooms to accumulate Hg, the edible wild-growing species accumulate the largest amount of Hg (Falandysz et al. 2015b; Melgar et al. 2009).

**Fig. 1:**
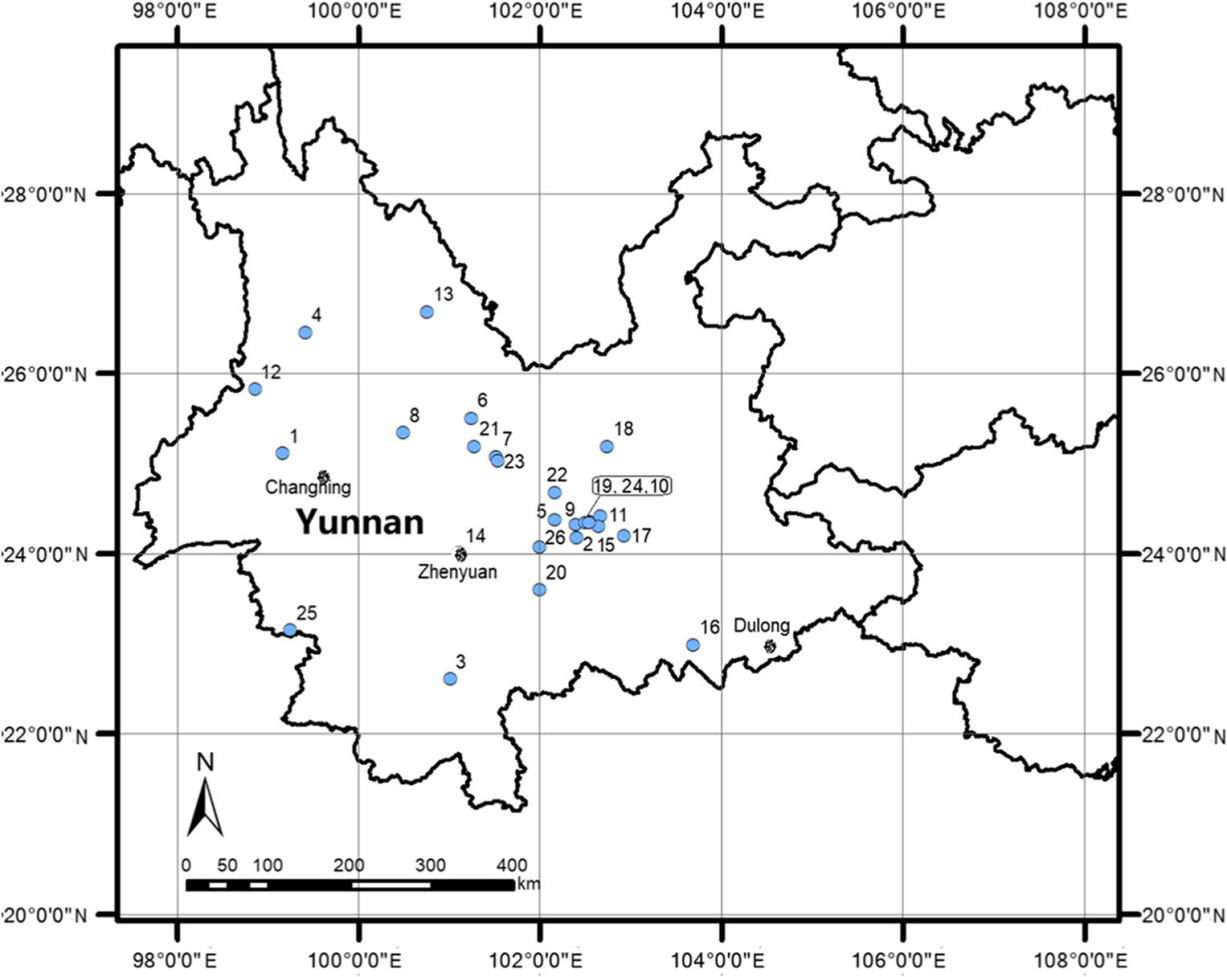
Localization of the sampling sites of mushrooms and topsoil from Yunnan

**Fig. 2:**
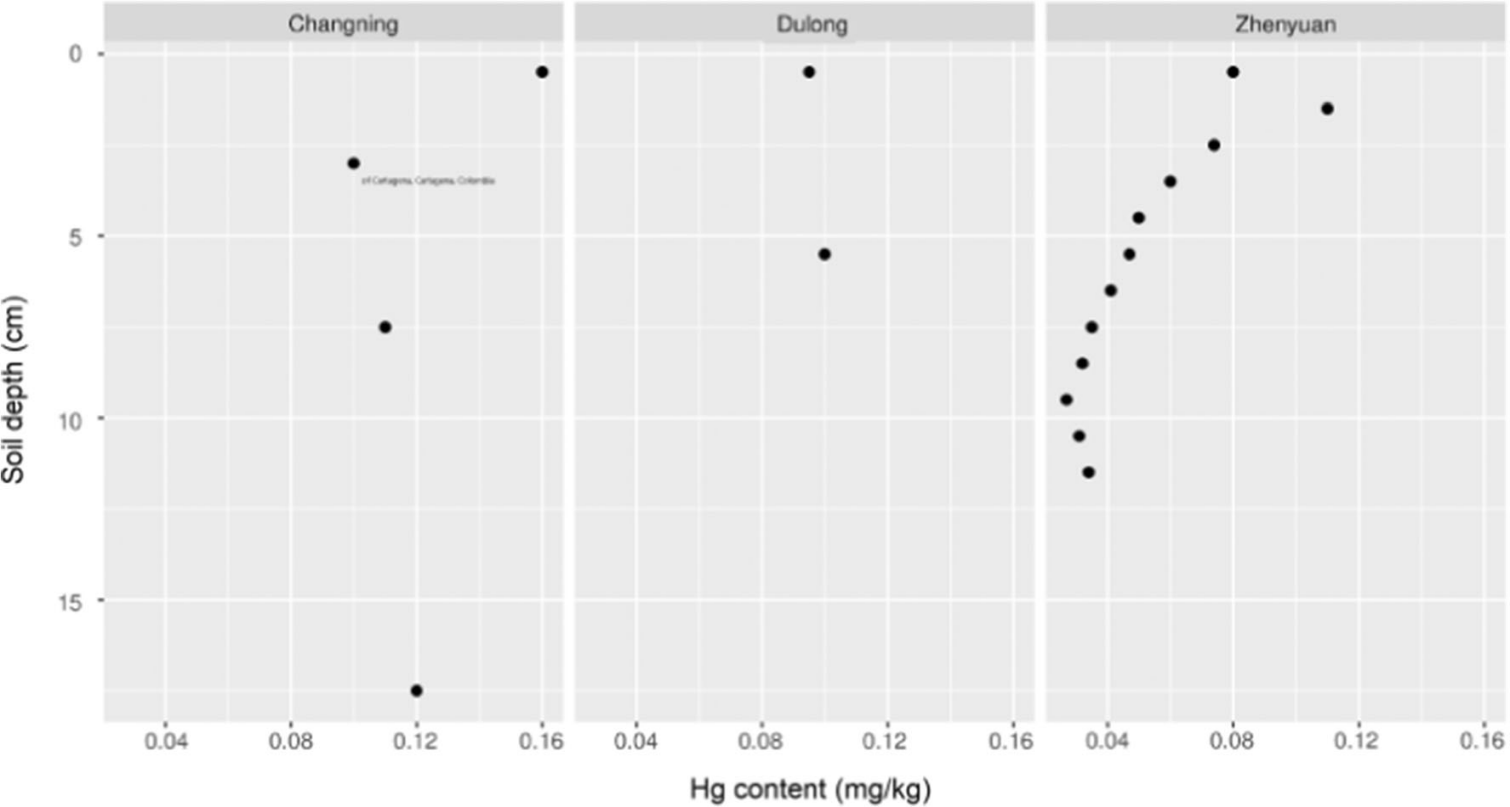
Mercury concentration (Hg mg kg^−1^ dw) profiles of the forest soil cores taken from Changning, Dulong, and Zhenyuan–Pu’er in the Yunnan province

The current study addresses this knowledge gap by investigating the fruiting bodies of 42 commercially important species of mushrooms from 23 sites in the Province of Yunnan collected during the period, 2011–2017 (Fig. 1). The collection sites were usually located in regions were mushrooms from the wild are intensively foraged, along with places where local markets for mushrooms exist. In parallel, samples of topsoil layers from three different forested locations spatially distantly distributed across the province (Figs. 1 and 2) were also collected in order to get some information on the Hg content in the soil substrata in Yunnan.

## Materials and methods

### Forest topsoil

Forest topsoil layers were collected in 2015 from undisturbed rural locations in Yunnan, at the Zhenyuan site (coordinates: 24°26′45″ N and 100°33′36″ E) at Mt. Ailao in the Pu’er prefecture at an altitude of circa 1400 m above sea level, from the Changning site (24°43′44″ N and 99°45′4″ E) at an altitude of 1950 m a.s.l., and from the Dulong site (22°53′21″ N and 104°32′52″ E) at an altitude of 915 m a.s.l. in 2016 (Figs. 1 and 2).

The soil layers at the sites were taken from different depth (Fig. 2) using a stainless steel slicer after the soil horizon was uncovered. Litter covering soil surface was removed before sampling. The sections of soil horizons sampled weighed circa 100 g each. If necessary, soils were freed from visible plant roots on site, and placed into brand new sealed polyethylene bags. At the laboratory, samples were air dried at room temperature for 8–10 weeks under dry and clean conditions to obtain aerially dry material. Dried soils were powdered in a porcelain mortar, sieved through a pore size of a 2-mm plastic sieve (a plastic sieve each time washed, well rinsed with deionized water, and dried before re-use), transferred into brand new sealed polyethylene bags, and kept until analysis for up to 1 week in tightly closed plastic containers under dry condition.

### Fungi

The wild mushroom species included genera such as *Baorangia*, *Boletus*, *Butyriboletus*, *Caloboletus*, *Catathelasma*, *Fomes*, *Fomitopsis*, *Ganoderma*, *Ischnoderma*, *Lactarius*, *Leccinum*, *Lentinus*, *Neoboletus*, *Retiboletus*, *Rubroboletus*, *Suillellus*, *Suillus*, *Sutorius*, *Thelephora*, *Tylopilus*, *Tyromyces*, and *Xerocomus* sp. In total, 42 species of medicinal edible fungi were collected, including: *Agaricus blazei* Murrill, *Baorangia bicolor* (Kuntze) G. Wu, Halling & Zhu L. Yang, *Boletus auripes* Peck, *Boletus bainiugan* Dentinger, *Boletus calopus* (Pers.) Vizzini, *Boletus speciosus* Frost, *Boletus tomentipes* Earle, *Butyriboletus regius* (Krombh.) D. Arora & J.L. Frank, *Butyriboletus subsplendidus* (W.F. Chiu) Kuan Zhao, G. Wu & Zhu L. Yang, *Caloboletus calopus* (Pers.) Vizzini, *Catathelasma ventricosum* (Peck) Singer, *Fomes rufolaccatus* Lloyd, *Fomitopsis pinicola* (Swartz.:Fr), *Fomitopsis ulmaria* (Sor.:Fr.) Bond. et Sing., *Ganoderma applanatum* (Pers.) Pat, *Ganoderma capense* (Lloyd) Teng, *Ganoderma japonicum* (Fr.) Lloyd, *Ganoderma kunmingense* Zhao, *Ganoderma lingzhi* Sheng H.Wu, Y.C.Dai & Y.Cao, *Ganoderma lucidum* (Leyss. ex Fr.), *Ganoderma luteomarginatum* Zhao, Xu et Zhang, *Ganoderma philippii* (Bres. & Henn. ex Sacc.) Bres, *Ganoderma ramosissimum* Zhao, *Ganoderma sinense* Zhao, Xu et Zhang, *Ganoderma tsugae* Murr, *Ischnoderma resinosum* (Schaeff.: Fr.) Karst., *Lactarius deliciosus* (L.) Gray, *Leccinum extremiorientale* (Lj.N. Vassiljeva) G. Wu & Zhu L. Yang, *Lentinula edodes* (Berk.) Pegler, *Neoboletus brunneissimus* (W.F. Chiu) G. Wu & Zhu L. Yang, *Retiboletus griseus* (Frost) Manfr. Binder & Bresinsky, *Retiboletus ornatipes* (Peck) Manfr. Binder & Bresinsky, *Rubroboletus sinicus* (W.F. Chiu) Kuan Zhao & Zhu L. Yang, *Suillellus luridus* (Schaeff.) Murrill, *Suillus bovinus* (L.) Roussel, *Sutorius magnificus* (W.F. Chiu) G. Wu & Zhu L. Yang, *Thelephora ganbajun* Zang, *Tylopilus chromapes* (Frost) A.H. Sm. & Thiers, *Tylopilus felleus* (Bull.:Fr.) Karst, *Tyromyces albidus* (Schaeff ex Secr.) Donk, and *Xerocomus* sp. The composite samples (*n* > 1) contained up to 100 individual fruiting bodies per species (Table 1) and location (Fig. 1).

**Table 1:** Contents of Hg (mg kg^−1^ dry weight; arithmetic mean and standard deviation) in the wild-growing and cultivated medicinal mushrooms from China

Species and year	Sampling site	Hg			*Q*_C/S_
		Caps	Stipes	Whole fruiting bodies^b^	
*Agaricus blazei* (100)^*a^ 2015	Longyang, Baoshan [1^#^]	0.085 ± 0.001	0.042 ± 0.001	0.064	2.0
*Amauroderma bataanense* (15) 2014	Eshan, Yuxi [2]	0.065 ± 0.001	0.041 ± 0.001	WD	1.6
*Amauroderma guangxiense* (/) 2011	Laiyanghe, Pu′er [3]	WD	WD	0.041 ± 0.001	WD
*Amauroderma niger* (/) 2012	Lanping, Nujiang [4]	WD	WD	0.24 ± 0.00	WD
*Baorangia bicolor* (56) 2017	Tadian, Yuxi [5]	3.1 ± 0.1	1.3 ± 0.0	2.2	2.4
*Baorangia bicolor* (13) 2017	Yaoan, Chuxiong [6]	5.0 ± 0.0	2.7 ± 0.0	3.9	1.8
*Baorangia bicolor* (5) 2017	Donggua, Chuxiong [7]	2.8 ± 0.1	1.3 ± 0.1	2.1	2.1
*Baorangia bicolor* (9) 2017	Midu, Dali [8]	0.76 ± 0.04	0.37 ± 0.02	0.57	2.0
*Baorangia bicolor* (22) 2017	Luohe, Yuxi [9]	8.6 ± 0.4	3.6 ± 0.2	6.1	2.4
*Baorangia bicolor* (5) 2017	Donggua, Chuxiong [7]	2.8 ± 0.1	1.3 ± 0.1	2.1	2.1
*Baorangia bicolor* (18) 2017	Liujie, Yuxi [10]	4.9 ± 0.2	4.3 ± 0.2	4.6	1.1
*Baorangia bicolor* (43) 2017	Midu, Dali [8]	1.5 ± 0.1	0.91 ± 0.03	1.2	1.6
*Boletus auripes* (12) 2017	Tadian, Yuxi [5]	0.93 ± 0.02	0.58 ± 0.00	0.76	1.6
*Boletus bainiugan* (14) 2017	Yaoan, Chuxiong [6]	5.6 ± 0.2	2.8 ± 0.1	4.2	2.0
*Boletus bainiugan* (6) 2017	Longyang, Baoshan [1]	6.1 ± 0.3	2.1 ± 0.1	4.1	2.9
*Boletus bainiugan* (22) 2017	Donggua, Chuxiong [7]	14 ± 0	6.0 ± 0.2	10	2.3
*Boletus bainiugan* (11) 2017	Longyang, Baoshan [1]	11 ± 0	4.8 ± 0.0	7.9	2.3
*Boletus bainiugan* (18) 2017	Midu, Dali [8]	1.0 ± 0.0	0.64 ± 0.03	0.82	1.6
*Boletus speciosus* (23) 2017	Longyang, Baoshan [1]	5.3 ± 0.2	3.2 ± 0.1	4.3	1.7
*Boletus speciosus* (2) 2017	Tadian, Yuxi [5]	2.3 ± 0.1	1.5 ± 0.0	1.9	1.5
*Boletus speciosus* (273) 2017	Longyang, Baoshan [1]	6.4 ± 0.1	4.0 ± 0.1	5.2	1.6
*Boletus speciosus* (15) 2017	Liujie, Yuxi [10]	4.8 ± 0.0	1.6 ± 0.1	3.2	3.0
*Boletus speciosus* (12) 2017	Luohe, Yuxi [9]	5.4 ± 0.3	3.6 ± 0.1	4.5	1.5
*Boletus tomentipes* (21) 2017	Liujie, Yuxi [10]	5.6 ± 0.1	1.1 ± 0.4	3.4	5.1
*Butyriboletus regius* (9) 2017	Luohe, Yuxi [9]	4.6 ± 0.1	2.3 ± 0.1	3.5	2.0
*Butyriboletus subsplendidus* (10) 2017	Longyang, Baoshan [1]	12 ± 1	5.2 ± 0.0	8.6	2.3
*Caloboletus calopus* (11) 2017	Longyang, Baoshan [1]	5.9 ± 0.0	1.7 ± 0.1	3.8	3.5
*Caloboletus calopus* (10) 2017	Yaoan, Chuxiong [6]	4.1 ± 0.1	1.1 ± 0.0	2.6	3.7
*Caloboletus calopus* (4) 2017	Longyang, Baoshan [1]	2.9 ± 0.0	1.2 ± 0.0	2.1	2.4
*Caloboletus calopus* (13) 2017	Anhua, Yuxi [11]	1.6 ± 0.1	1.1 ± 0.0	1.4	1.5
*Caloboletus calopus* (11) 2017	Longyang, Baoshan [1]	5.9 ± 0.0	1.7 ± 0.1	3.8	3.5
*Caloboletus calopus* (10) 2017	Luohe, Yuxi [9]	9.8 ± 0.3	8.4 ± 0.0	9.1	1.2
*Caloboletus calopus* (3) 2017	Tadian, Yuxi [5]	1.3 ± 0.0	0.70 ± 0.00	1.0	1.9
*Catathelasma ventricosum* (6) 2014	Eshan, Yuxi [2]	5.5 ± 0.0	1.4 ± 0.1	3.9	3.9
*Fomes rufolaccatus* (1) 2014	Zhengyuan, Pu′er [14]	WD	WD	0.069 ± 0.001	WD
*Fomes rufolaccatus* (1) 2014	Jiuxi, Yuxi [15]	WD	WD	0.12 ± 0.00	WD
*Fomitopsis pinicola* (1) 2014	Lushui, Nujiang [12]	WD	WD	0.024 ± 0.001	WD
*Fomitopsis pinicola* (1) 2014	Lushui, Nujiang [12]	WD	WD	0.027 ± 0.000	WD
*Fomitopsis pinicola* (1) 2014	Yongsheng, Lijiang [13]	WD	WD	0.035 ± 0.002	WD
*Fomitopsis pinicola* (1) 2014	Lushui, Nujiang [12]	WD	WD	0.018 ± 0.001	WD
*Fomitopsis ulmaria* (1) 2014	Lanping, Nujiang [4]	WD	WD	0.13 ± 0.00	WD
*Ganoderma applanatum* (/) 2012	Lanping, Nujiang [4]	WD	WD	0.11 ± 0.01	WD
*Ganoderma capense* (/) 2012	Pingbian, Honghe [16]	WD	WD	0.19 ± 0.01	WD
*Ganoderma japonicum* (10) 2014	Huaning, Yuxi [17]	0.088 ± 0.002	0.047 ± 0.00	0.080	1.9
*Ganoderma kunmingense* (15) 2012	Xiaoshao, Kunming [18]	WD	WD	0.012 ± 0.002	WD
*Ganoderma lingzhi* (8) 2013	Xiaoshao, Kunming [18]	0.086 ± 0.004	0.088 ± 0.00	0.087	0.98
*Ganoderma lingzhi* (25) 2014	Xiaoshao, Kunming [18]	0.10 ± 0.00	0.077 ± 0.003	0.095	1.3
*Ganoderma lingzhi* (17) 2015	Xiaoshao, Kunming [18]	0.088 ± 0.00	0.042 ± 0.002	0.079	2.1
*Ganoderma lingzhi* (/) 2012	Dayingjie, Yuxi [19]	WD	WD	0.096 ± 0.004	WD
*Ganoderma lingzhi* (6) 2013	Yuanjiang, Yuxi [20]	0.10 ± 0.00	0.081 ± 0.004	0.096	1.2
*Ganoderma lingzhi* (10) 2013	Lanping, Nujiang [4]	0.086 ± 0.00	0.053 ± 0.002	0.079	1.6
*Ganoderma lucidum* (7) 2012	Huaning, Yuxi [17]	0.085 ± 0.001	0.074 ± 0.004	0.083	1.1
*Ganoderma luteomarginatum* (10) 2012	Huaning, Yuxi [17]	0.18 ± 0.00	0.095 ± 0.003	0.16	1.9
*Ganoderma philippii* (10) 2011	Laiyanghe, Pu′er [3]	WD	WD	0.022 ± 0.001	WD
*Ganoderma philippii* (/) 2011	Laiyanghe, Pu′er [3]	WD	WD	0.033 ± 0.001	WD
*Ganoderma ramosissimum* (10) 2012	Huaning, Yuxi [17]	WD	WD	0.059 ± 0.003	WD
*Ganoderma sinense* (8) 2013	Lanping, Nujiang [4]	WD	WD	0.20 ± 0.00	WD
*Ganoderma tsugae* (/) 2012	Lanping, Nujiang [4]	WD	WD	0.085 ± 0.002	WD
*Ischnoderma resinosum* (1) 2014	Nanhua, Chuxiong [21]	WD	WD	0.060 ± 0.000	WD
*Lactarius delicious* (30) 2017	Longyang, Baoshan [1]	1.2 ± 0.0	0.83 ± 0.04	1.1	1.4
*Leccinum extremiorientale* (23) 2017	Yimen, Yuxi [22]	6.1 ± 0.5	2.2 ± 0.1	3.6	2.8
*Leccinum extremiorientale* (12) 2017	Luohe, Yuxi [9]	3.7 ± 0.1	1.2 ± 0.0	2.1	3.1
*Leccinum extremiorientale* (10) 2017	Donggua, Chuxiong [7]	3.3 ± 0.1	1.4 ± 0.2	2.1	2.4
*Leccinum extremiorientale* (9) 2017	Luohe, Yuxi [9]	3.6 ± 0.2	1.3 ± 0.1	2.1	2.8
*Lentinula edodes* (70) 2015	Wuding, Chuxiong [22]	0.039 ± 0.002	0.029 ± 0.001	0.034	1.3
*Lentinula edodes* (100) 2015	Longyang, Baoshan [1]	0.015 ± 0.001	0.015 ± 0.001	0.015	1.0
*Neoboletus brunneissimus* (6) 2017	Liujie, Yuxi [10]	2.8 ± 0.0	0.95 ± 0.03	1.9	2.9
*Retiboletus griseus* (46) 2017	Zixi, Chuxiong [23]	1.1 ± 0.0	0.72 ± 0.01	0.91	1.5
*Retiboletus griseus* (10) 2017	Dayingjie, Yuxi [19]	2.3 ± 0.0	1.4 ± 0.1	1.8	1.6
*Retiboletus ornatipes* (10) 2017	Liujie, Yuxi [10]	1.4 ± 0.0	1.5 ± 0.1	1.5	0.93
*Retiboletus ornatipes* (9) 2017	Donggua, Chuxiong [7]	2.8 ± 0.2	1.0 ± 0.0	1.9	2.8
*Rubroboletus sinicus* (4) 2017	Liujie, Yuxi [10]	2.6 ± 0.1	3.1 ± 0.0	2.9	0.68
*Suillellus luridus* (7) 2017	Liujie, Yuxi [10]	2.6 ± 0.0	1.1 ± 0.0	1.9	2.4
*Suillus bovinus* (8) 2017	Hongta, Yuxi [24]	0.4 ± 0.0	0.07 ± 0.01	0.30	5.7
*Sutorius magnificus* (19) 2017	Midu, Dali [8]	4.3 ± 0.3	1.8 ± 0.0	3.0	2.4
*Sutorius magnificus* (6) 2017	Tadian, Yuxi [5]	4.1 ± 0.0	2.8 ± 0.1	3.4	2.3
*Sutorius magnificus* (6) 2017	Liujie, Yuxi [10]	2.0 ± 0.0	1.6 ± 0.1	1.8	1.2
*Thelephora ganbajun* (1) 2013	Cangyuan, Lincang [25]	WD	WD	0.13 ± 0.00	WD
*Thelephora ganbajun* (1) 2014	Jiuxi, Yuxi [15]	WD	WD	0.13 ± 0.02	WD
*Tylopilus chromapes* (32) 2017	Tadian, Yuxi [5]	3.3 ± 0.0	2.0 ± 0.0	2.7	1.6
*Tylopilus felleus* (5) 2017	Donggua, Chuxiong [7]	7.2 ± 0.4	3.1 ± 0.0	5.1	2.3
*Tyromyces albidus* Donk. (1) 2014	Ailao Mountain, Yuxi [26]	WD	WD	0.024 ± 0.01	WD
*Tyromyces albidus* Donk. (1) 2014	Jiuxi, Yuxi [15]	WD	WD	0.046 ± 0.001	WD
*Xerocomus* sp. (8) 2017	Luohe, Yuxi [9]	6.8 ± 0.3	5.1 ± 0.1	6.1	1.3

*WD* without data

*Number of fruiting bodies in a composite sample

/Number of fruiting bodies unknown

^a^Cultivated

^#^ID of a site (see Fig. 1)

At collection sites, mushrooms were cleaned from any visible foreign matter using a ceramic knife and a plastic brush. For some species, the whole fruiting bodies were used and for others, each individual fruiting body was separated into cap and stipe, and the individual samples were pooled accordingly (Table 1). The fungal materials were dried at 65 °C to constant mass (Ultra FD1000 dehydrator, Ezidri, Australia) (Kojta et al. 2015). The dried, fungal materials were ground in a porcelain mortar to a fine powder, passed through an 80-mesh sieve and stored in a screw-sealed plastic (low-density polyethylene) bags under dry conditions.

### Determination of Hg

The analytical methodology for Hg determination in fungal and soil materials has been presented in detail before but a summarized description is given below (Jarzyńska and Falandysz 2011; Saba et al. 2016c). Double distilled water was used for the preparation of the solutions. A mercury standard solution of 1.0 mg Hg mL^−1^ was obtained from the 10 mg mL^−1^ standard stock solution (Merck). Blanks and 25, 50, 100, 150, and 200 μL (high mode) and 3, 5, 10, 15, and 20 μL (low mode) of 1.0 mg mL^−1^ Hg standard solutions were injected into the Hg analyzers for the construction of calibration curves.

The determinations of Hg content were performed using cold-vapor atomic absorption spectroscopy (CV-AAS) by direct sample thermal decomposition coupled with gold wool trap and amalgamation of Hg vapor and Hg desorption and quantitative measurement at a wavelength of 253.7 nm. The instruments used were the MA-2000 mercury analyzers (Nippon Instruments Corporation, Takatsuki, Japan), both, with and without auto sampler and operated respectively in high (25 to 150 ng Hg per sample) and low (3 to 20 ng Hg per sample) modes (Jarzyńska and Falandysz 2011).

Quality assurance and quality control measures included the analysis of procedural blanks and certified reference materials as described before (Falandysz et al. 2015b; Jarzyńska and Falandysz 2011; Saba et al. 2016c). Two certified reference materials were analyzed: CS-M-2 (dried *Agaricus campestris*) with declared concentration of Hg at 0.164 ± 0.004 mg kg^−1^ and determined concentration of Hg at 0.16 ± 0.01 mg kg^−1^ (*n* = 8; recovery 98%), and CS-M-3 (dried *Boletus edulis*) with declared concentration of Hg at 2.849 ± 0.104 mg kg^−1^ and determined concentration of Hg at 2.8 ± 0.0 mg kg^−1^ (*n* = 5; recovery 98%). The number of replicates was 2–3 for the material with relatively high Hg content, while 2–4 for the material with relatively low Hg content. Two blank samples were included with each sample studied and four subsamples of certified reference materials were analyzed with each set of 8 real samples.

## Results

### Hg in forest topsoil

The Hg concentration in forest topsoil at different depths from three sites spatially scattered across the Yunnan province suggests that, both, geogenic sources corresponding to specific topsoil profiles of Hg in Dulong and Changning, and an anthropogenic influence in Changning and Zhenyuan, seem possible (Fig. 2). A substantial enrichment of Hg in the top 0-–6.0-cm layer and especially in the organic 0-–4.0-cm layer (Hg contents from 0.060 to 0.11 mg kg^−1^ dry weight (dw) in segments) of forest topsoil near the Zhenyuan village in Mt. Ailao region is clear.

Mercury contents in segments of the mineral horizon in the Zhenyuan site were in the range 0.027 to 0.050 mg kg^−1^ dw in 5.0- to 12-cm layer, which is around a half of the results for Changning (0.10 to 0.12 mg kg^−1^ dw in section of 1.0 to 25 cm) and Dulong (0.10 mg kg^−1^ dw in section of 1.0 to 10 cm). A geogenic source of Hg at the Changning and Dulong sites seems clear (Figs. 1 and 2), and isotopic fingerprints could further aid the identification of sites with Hg geological anomalies where wild mushrooms are traditionally harvested in Yunnan. A weak organic layer of topsoil from the forested area near the Changning site was relatively thin (0–1.0 cm) and was almost absent in the profile from the Dulong site.

### Hg in fungi

In this study, species of wood-decaying macromycetes growing on the trunks of living or dead trees included species such as *Amauroderma bataanense*, *Amauroderma guangxiense*, *Amauroderma niger*, *F. rufolaccatus*, *F. pinicola*, *F. ulmaria*, *G. applanatum*, *G. capense*, *G. japonicum*, *G. kunmingense*, *G. lingzhi*, *G. lucidum*, *G. luteomarginatum*, *G. philippii*, *G. ramosissimum*, *G. sinense*, *G. tsugae*, *I. resinosum*, and *L. edodes*. The stipes contained Hg in the range from 0.015 ± 0.001 in *L. edodes* to 0.095 ± 0.003 mg kg^−1^ dw in *G. luteomarginatum*, while cap concentrations ranged from 0.015 ± 0.001 in *L. edodes* to 0.18 ± 0.00 mg kg^−1^ dw in *G. luteomarginatum*, and the whole fruiting bodies, from 0.015 in *L. edodes* to 0.19 ± 0.01 mg kg^−1^ dw in *G. capense* (Table 1). The maximum concentration of Hg between the polypore species in this study was 0.24 mg kg^−1^ dw in a sample of *A. niger*. Data for these wood-decaying fungi usually showed higher content of Hg in caps than stipes. The cap to stipe concentration quotient (*Q*_C/S_) was in the range 0.98 to 2.1 (median 1.4) for *G. lingzhi*, from 1.1 to 1.9 for other *Ganoderma* mushrooms, from 1.0 to 1.3 for *L. edodes*, and 1.6 for *A. bataanense* (Table 1).

In addition to wood-decaying wild mushrooms, the other types investigated in this study were the ectomycorrhizal species such as *B. bicolor*, *B. bainiugan*, *B. speciosus*, *B. regius*, *B. subsplendidus*, *C. calopus*, *C. ventricosum*, *L. extremiorientale*, *N. brunneissimus*, *R. griseus*, *R. ornatipes*, *R. sinicus*, *S. luridus*, *S. magnificus*, *T. chromapes*, *T. felleus*, and *Xerocomus* sp., which all showed Hg concentrations from one to three orders of magnitude higher (Table 1) than the wood-decaying species studied. The only non-wood-decaying saprobic species studied was *A. blazei*, which showed Hg in a concentration similar to those determined in the wood-decaying species.

Some of the highest Hg concentrations found were in species such as *B. bicolor* (up to 8.6 ± 0.4 mg kg^−1^ dw), *B. bainiugan* (up to 14 ± 0 mg kg^−1^ dw), *B. speciosus* (up to 6.4 ± 0.1 mg kg^−1^ dw), *B. regius* (up to 4.6 ± 0.1 mg kg^−1^ dw), *B. subsplendidus* (up to 12 ± 1 mg kg^−1^ dw), *C. calopus* (up to 9.8 ± 0.3 mg kg^−1^ dw), *S. magnificus* (up to 4.3 ± 0.3 mg kg^−1^ dw), *C. ventricosum* (up to 5.5 ± 0.0 mg kg^−1^ dw), *L. extremiorientale* (up to 6.1 ± 0.5 mg kg^−1^ dw), *T. felleus* (up to 7.2 ± 0.4 mg kg^−1^ dw), or *Xerocomus* sp. (up to 6.8 ± 0.3 mg kg^−1^ dw), while usually stipes showed from 1.2- to 5.7-fold (Table 1) lower than the caps.

The quality of the medicinal mushrooms in this study in view of contamination with Hg presents a complex picture both due to the kind of species collected and the difference in the pattern of contamination between them. The medicinal species including all the “hard” type polypore fungi, *L. edodes* and *T. ganbajun*, presented a lower risk as far as the possible toxic effects of total Hg could be considered (Table 1). As mentioned, Hg concentrations in these mushrooms were up to 0.24 mg kg^−1^ dw, as seen in *A. niger*. Hence, consumer exposure to Hg contained in the hypothetical medicinal derivatives of these mushrooms was investigated alone for the “hard” species including *L. edodes* and *T. ganbajun* using a maximum value of 0.24 mg kg^−1^dw and separately for other soft flesh mushrooms—largely from the *Boletaceae* family, using a maximum value of up to 10 mg kg^−1^ dw for a whole fruiting body of *B. bainiugan* from the Donggua site in the Chuxiong region of Yunnan (Table 1).

### Estimated intake of Hg

The exposure estimations in this study assumed an intake biomass of 3 × 0.5 g of capsulated dried product—both of “hard” and “soft” flesh type mushrooms per capita daily over a week, and from 100 to 300 g (maximal 500 g) in a meal (*Boletaceae* family species). The intakes also assumed the maximal Hg concentrations for “hard” and “soft” flesh type mushrooms, respectively, for an adult Asian individual (60 kg body mass, bm). The “worst case” estimates showed intake of Hg from 0.006 μg kg^−1^ bm (“hard” type polypore) and 0.25 μg kg^−1^ bm (“soft” flesh *Boletaceae*) on a daily basis for capsulated products, and 17, 50, and 83 μg kg^−1^ bm in the case of meals made of *Boletaceae* mushrooms, respectively. Hence, on a weekly basis, intake could reach 0.042, 1.7, 120, 350, and 580 μg kg^−1^ bm.

## Discussion

### Topsoil

In this study, the site of Zhenyuan represents a sparsely inhabited rural area with small farms scattered around and the region is remote from the urbanized and industrialized sites/centers of China. A recent study of Hg concentration and isotopic signatures in the forest topsoil (2016) and litter fall (2017) samples collected from the windward (1250–2400 m a.s.l.) and leeward slopes of Mt. Ailao (850–2400 m a.s.l) suggested that contamination of topsoil (0–10 cm layer) is largely from litter fall input, which relates well with the observation in this study (Fig. 2) (Fu et al. 2019).

In China soil, with Hg concentration ≤ 0.150 mg kg^−1^ dw could be qualified as grade I (uncontaminated) according to the Chinese Environmental Quality Standard for Soils (Shi et al. 2013). Nevertheless, a regional and local enrichment in Hg of forest topsoils in Yunnan suggests a strong contribution from the Hg background in bedrock (Nie et al. 2018).

Previous studies that analyzed soils collected underneath mushrooms sampled from the forests of Yunnan have found that the concentration of Hg in the top layer (0–10 cm) is elevated when compared with forest topsoils from the regions of southern and northern Europe. This type of soil from Croatia and northern parts of Poland typically shows Hg at concentration well below 0.05 mg kg^−1^ dw (Falandysz et al. 2015b; Širić and Falandysz 2020). For example, in China, the topsoils from 44 sites sampled in parallel with the *Boletaceae* family mushrooms from rural and anthropogenically unpolluted regions showed Hg in the range 0.034 to 3.4 mg kg^−1^ dw, and from 12 sites where *Leccinum* mushrooms were collected, in the range from 0.065 to 0.58 mg kg^−1^ dw (Falandysz et al. 2015a, b). In topsoil underneath *Xerocomus* spp. in Yunnan, the Hg concentration was in the range 0.21 to 0.49 mg kg^−1^ dw (*n* = 4) and topsoil underneath *Macrocybe gigantea* (Pu’er prefecture in Yunnan) was in the range 0.075 to 0.24 mg kg^−1^ dw (*n* = 7) (Kojta et al. 2015; Wiejak et al. 2014).

### Fungi

Wood-decaying polypore mushrooms and *L. edodes* have a special position in traditional herbal medicine in China, and species examined in this study showed low contamination with Hg. As far as possible toxic effects were concerned, the Hg concentrations observed in the studied *Ganoderma* and other wood-decaying mushrooms would imply a relatively low level of health concern.

Nowadays, formulations made for medicinal and gourmet mushrooms or cultivated medicinal and edible species can be bio-fortified with certain elements, e.g., selenized or lithiumized (often a composite of several species) and sold in capsules as a food supplement as a mix of dried and powdered products. It would be useful to know whether the manner of preparation (processing) of the medicinal mushrooms during formulation can have an effect on the Hg (and other elements) content of the final products but no data could be found in the available literature.

Preparation processes involve water or alcohol (ethanol) extracts or a composite of alcohol and water extracts of sliced, dried, or fresh, polypore mushrooms (reishi mushrooms, e.g., *G. lucidum*) from cold maceration or making a decoction by simmering with water at different time intervals (up to several weeks) and following a specific recipe. Therefore, a liquid formula obtained can be a simple macerate, tincture, decoct, or a composite elixir. A product can also be in a form of a powder prepared from a decoct after evaporation of the water.

Traditional culinary practices of preparing mushrooms such as stir frying in oil or braising do not decrease, while can increase contents, e.g., Hg, radiocaesium (^137^Cs) and natural ^40^K (hence also K) in mushroom meals, when related to the contents in the fresh mushrooms based on the whole (wet) weight (Falandysz et al. 2019a, b, 2020b, c). Hot water alone or with a chelating agent will extract a proportion of the mineral constituents including Hg from fungal flesh (Drewnowska et al. 2017a, b; Stijve 1994; Svoboda et al. 2002). For example, traditional blanching using only water and boiling removes some Hg together with water soluble compounds and colloids from fresh fruiting bodies, e.g., *Cantharellus cibarius* and *Amanita fulva* mushrooms (Falandysz and Drewnowska 2017), which implies that not only water extracts but also organic solvent extracts (MeHg is also lipophilic compound) can transfer Hg compounds from a mushroom substrate to formulations.

It must be pointed out that these reported values of Hg are within the ranges noticed in several other species of edible mushrooms from the regions of Yunnan. The maximum concentration recorded was in a composite sample of fruiting bodies of the *B. bainiugan* (called *B. edulis* in Yunnan before a molecular phylogenetic recognition as a new genus in the family of *Boletaceae*; *B. edulis* is absent in Yunnan) with 22 mg kg^−1^ dw in the caps, 8.4 mg kg^−1^ dw in the stipes (Falandysz et al. 2015b).

There is no data available on the extraction efficiency of Hg (all chemical forms of Hg) during the production of dried pure (medicinal grade) extracts from any polypore mushroom. A 15-min blanching of fresh mushrooms in tap water has limited effect on the release (loss) of Hg from fruiting bodies on a dry weight basis (Falandysz and Drewnowska 2015, 2017). Stir frying in deep oil can cause the increase in Hg concentration in fried mushrooms (Falandysz et al. 2019a, b) and braising the effect of braising was to increase the average total Hg (THg) and MeHg contents in fresh mushroom meals by around 50% respectively, but a reduction of around 40% respectively was seen on a dry weight basis (submitted). The increase in Hg concentration in fried or braised mushrooms can be explained by the partial decline in moisture content during processing and the resulting shrinkage of the fungal matrix accompanied by the preferential retention of a portion of Hg.

However, the polypore species used for medicines are first dried and pulverized and depending on the recipe can then be, e.g., macerated for a long period of time with ethanol (vodka) and water and finally, extracted, dried, and capsuled. Such a process of prolonged maceration and extraction could be more effective at removing Hg than blanching for a short period with hot or boiling tap water. Hence, due to the lack of data on Hg in dried pure extracts from polypore fungi that can be purchased in retail outlets, assessments of Hg intake could make the assumption that Hg concentration in a formulation is the same as that in the original substrate based on dry biomass. As mentioned, some fungal supplements are composed only of dried and powdered mushrooms bio-fortified with Se or Li, where the content of the bio-element is around 10-fold higher than in the fresh substrate. A gelatine capsule is filled with 500 mg of fungal formulation regardless of the size of the capsule. On the other hand, the quantity of cooked edible mushrooms in a single meal is estimated at 100 to 300 g and occasionally above 500 g. The frequency of eating wild mushrooms and hence also the annual rate of consumption varies between people, countries, and regions and availability during a particular year, and the maximum quantity reported was 20 kg fresh biomass in Yunnan of China and up to 26 kg in the UK (Barnett et al. 2001; Zhang et al. 2010). Because of their particular diet, vegans and vegetarians can be characterized by a higher rate of mushrooms as also can Asian people, where wild mushrooms form a popular meal although there are some individuals who cannot eat them. Mushrooms are a vitamin D–rich source in the diet of vegetarians and vegans (Kim et al. 2018), and are several dozen recipes for mushroom dishes intended for vegetarians and vegans.

Wood-decaying fungi, both of hard (polypore) and soft (*L. edodes*) types, were poorer accumulators of Hg compared with the ectomycorrhizal and saprobic species in this (Table 1) and other studies (Dryżałowska and Falandysz 2014; Melgar et al. 2009; Saba et al. 2016a, b). Comparatively, hard (polypore) species of wood-decaying macromycetes show lower K, Rb, and Na contents than soft species (Tyler 1982). They are also relatively low in other metallic elements and metalloids including Cd (Siwulski et al. 2017), but they seem to be useful as indicators of atmospheric pollution by Pb (Gabriel et al. 1997; Tyler 1982).

### Hg exposure assessment

The estimated maximal doses derived from data for the *Boletaceae* mushrooms exceed the published daily reference dose (RfD) for Hg at 0.3 μg kg^−1^ bm or the provisionally tolerable weekly intake (PTWI) that is 3.4 μg kg^−1^ bm for a 60 kg bm individual (derived from the original value of 4.0 μg kg^−1^ bm for a 70 kg bm individual) (WHO 2011).

The natural antagonists of Hg are selenium (Se) and sulfur (S), with Se being a specific antagonist for MeHg (Ralston and Raymond 2018). Neither Se and S were determined in this study, although they can play a role in the protection of a consumer from the excess Hg (and MeHg) contained in wild mushrooms, e.g., in mushrooms of some genera from the family *Boletaceae* rich in Se (Falandysz 2013; Kavčič et al. 2019; Nasr et al. 2012). There is no doubt that mushrooms from certain regions of the world are foods that can be elevated in Hg because of species physiology and in parallel with Hg geological anomalies in background regions (without substantial local pollution), and in a part due to global fallout (Falandysz et al. 2015b; Melgar et al. 2009; Ostos et al. 2015). Mushrooms can also efficiently pick up Hg from grounds contaminated with this element because of cinnabar mining or from other industrial sources (Árvay et al. 2014; Falandysz 2016; Kavčič et al. 2019).

A recent study showed high concentrations of Se in mushrooms from Yunnan such as *B. bicolor*, *B. bainiugan*, *B. roseoflavus*, *R. griseus*, *R. extremiorientalis*, and *S. magnificus*. Concentrations ranged from 5.2 to 56 mg kg^−1^ dw, with stir-fried mushroom meals in the range from 4.6 mg kg^−1^ dw for *R. extremiorientalis* to 33 mg kg^−1^ dw for *B. roseoflavus* (Falandysz et al. 2020b). In the evolutionary process of minerals bio-geo-cycling, Hg and Se can interact, and ingested MeHg, which is highly neurotoxic, can interact with Se via selenoenzyme in neuronal cells in the central nervous system causing irreversible inhibition of selenium (Se)-dependent enzymes which are protective of oxidative damage in brain cells. The occurrence of Se in molar excess of MeHg counters this interruption of selenoenzyme activities (Ralston and Raymond 2018). The contents of Se both in some mushrooms from the *Boletaceae* family and in stir-fried meals made from them largely exceed (on a molar basis) the co-occurring Hg concentrations (Falandysz et al. 2020b). Thus, an excess of bioaccessible Se co-occurrence may not create susceptibility to the neurotoxic effect from the different forms of Hg in these mushrooms for exposed humans. Curiously, the sclerotia of medicinal fungus *Wolfiporia cocos* (Schwein.) Ryvarden & Gilb., from Yunnan, contained Hg concentrations (molar basis) that exceeded Se (Falandysz et al. 2020a). The incidence of polymetallic belts, Hg geochemical anomalies, substantially elevated concentrations of Hg in mushroom meals combined with relatively high intake rates in Yunnan, calls for further studies on this topic.

“Hard” type medicinal polypore fungi in Yunnan province in China showed relatively low contamination with Hg while “soft” types which are used both as edible and medicinal fungi (e.g., from the family *Boletaceae*) showed from one to three orders of magnitude higher concentrations which implies substantial contamination. The Hg concentrations in forest topsoil layers showed localized influences both from geogenic background and airborne deposition (probably largely from litter fall). The estimated intake of Hg from medicinal fungi of Yunnan based on data for a raw substrate can be considered low in the case of polypore species, but could be substantial in the case of “soft” type fungi from the family *Boletaceae*. There is a huge gap in knowledge on possible effect of the technologies used in processing of fungi on the Hg content in medicinal formulations.
